# Editorial: Nanotechnology and bioengineering platforms for drug and gene delivery

**DOI:** 10.3389/fbioe.2023.1328961

**Published:** 2023-11-23

**Authors:** Fanyi Mo, Jun Liu, Gang Chen, Ningqiang Gong, Zhaoting Li

**Affiliations:** ^1^ Pharmaceutical Sciences Division, School of Pharmacy, University of Wisconsin-Madison, Madison, WI, United States; ^2^ Key Laboratory of Marine Drugs, Chinese Ministry of Education, School of Medicine and Pharmacy, Ocean University of China, Qingdao, China; ^3^ School of Rehabilitation Sciences and Engineering, University of Health and Rehabilitation Sciences, Qingdao, China; ^4^ Department of Bioengineering, University of Pennsylvania, Philadelphia, PA, United States; ^5^ School of Medicine, The Chinese University of Hong Kong, Shenzhen, Guangdong, China

**Keywords:** nanotechnology, bioengineering, drug delivery, gene delivery, precision medicine

## 1 Introduction

Drug and gene delivery is a progressed area in the contemporary medical landscape, eliciting heightened clinical relevance and bringing new hopes for the treatment of intractable diseases. As therapeutic approaches evolve toward precision medicine, there is an imperative need for integrated delivery systems to allow for controlled drug administration and precise delivery (Guo and Szoka, 2003). However, drug and gene delivery still face a plethora of challenges, which range from inadequate bioavailability and targeting accuracy to the rapid degradation of therapeutic agents and potential side effects. More importantly, they also need to overcome physiological barriers such as the blood-brain barrier, combat multi-drug resistance in cancer cells, and secure stable, efficient delivery mechanisms. Traditional methods encounter their own set of limitations, particularly cellular toxicity, which can undermine therapeutic efficacy and compromise patient safety. Given these challenges, the fields of nanotechnology and bioengineering are being explored to develop refined, adaptable, and efficacious platforms for delivering drugs and genes to achieve precision medicine and personalized therapy (Pardridge, 2002; Sideratou et al., 2006; Lee and Park, 2018).

**FIGURE 1:**
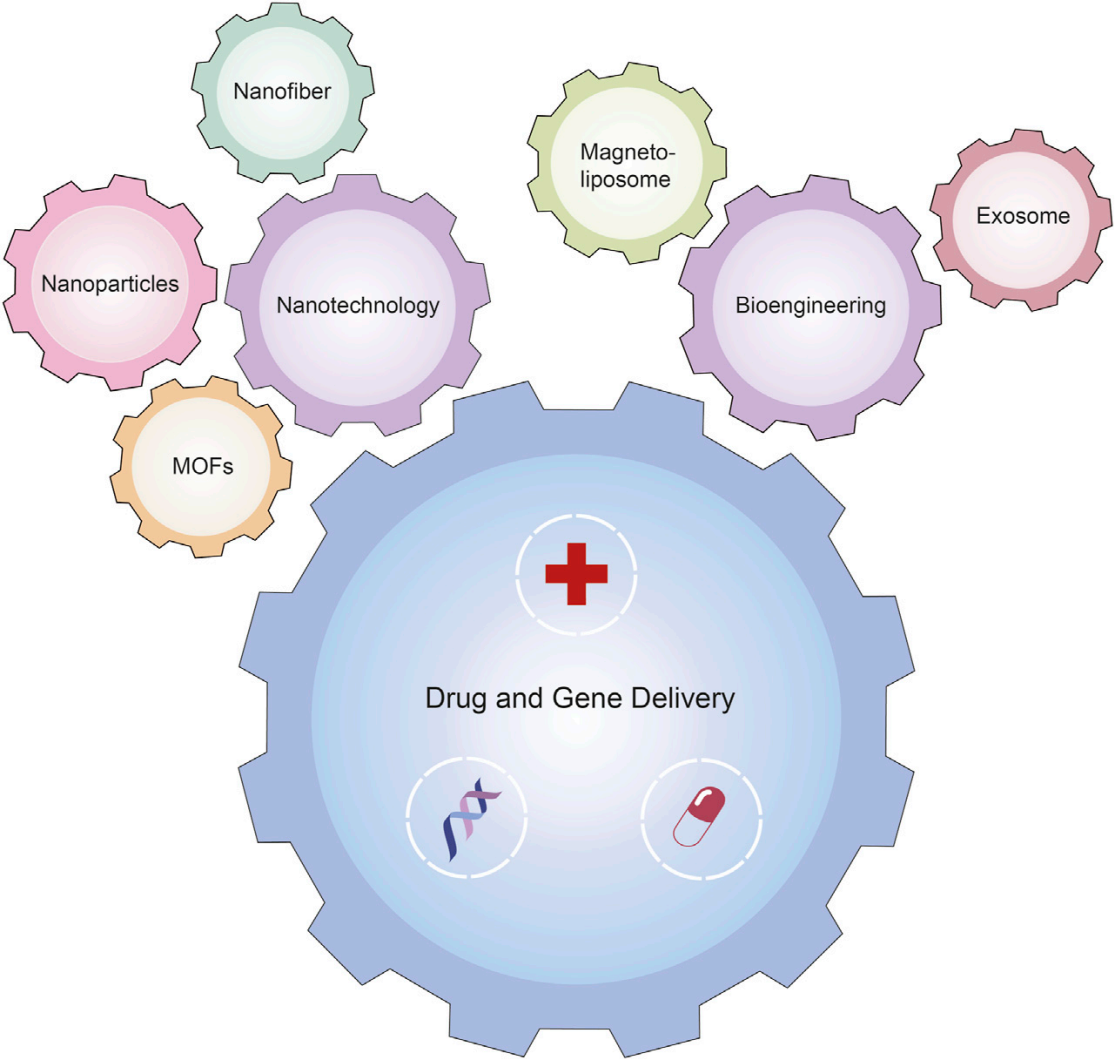
Schematic illustration of the nanotechnology and bioengineering platforms for drug and gene delivery.

The most recent advancements in nanotechnology and bioengineering endeavor to surmount prevailing limitations, such as the key challenges of systemic toxicity and physiological barriers, by providing capabilities for precision targeting, regulated drug release, and the realization of synergistic drug co-loading. These enhancements are pivotal for improving biocompatibility and optimizing cellular uptake, thus addressing the multifaceted challenges in medical interventions (Figure 1).

## 2 Nanotechnology for drug and gene delivery

### 2.1 Nanoparticle based drug delivery

Nanoparticles are ultrafine particles with dimensions measured in nanometers. They exhibit unique physical and chemical properties that differ from those of bulk materials due to their size. These characteristics make nanoparticles been wildly used in various fields, especially in drug delivery, where they can enhance drug absorption, prolong drug action, and guide drugs to specific tissues or cells (Cabral et al., 2011). In the nanotechnology-based platforms for drug and gene delivery, nanoparticles are vital for co-delivery. They’re designed to simultaneously deliver multiple therapies, targeting varied disease facets for enhanced treatment. Several studies indicate the promise of using nanoparticles for co-delivery and complex treatments. For instance, layer-by-layer nanoparticles were utilized to co-deliver 5-Fluorouracil with gene regulators, demonstrating enhanced drug stability and reduced tumor growth, and offering an effective path for improved colorectal cancer treatment efficacy (Shahidi et al.). Another notable research by Li et al. introduced MitoQ@PssL NPs, a nanoparticle system designed specifically for treating periodontitis. The system encapsulates mitoquinone (MitoQ) known for enhancing autophagy. When deployed in the ROS-rich environment characteristic of periodontitis, the ROS-cleavable polymer PssL disintegrates, leading to the release of MitoQ. This system offers targeted release, potentially lowering toxicity and improving current treatments especially for periodontitis and other similar diseases (Li et al.).

Furthermore, Yang et al. presented a nano-vaccine that could precisely target and enhance the delivery to lymphoid organs, showing significant antitumor effects and reduced immunotoxic side effects (Yang et al.). Besides the application in nano-vaccine, Hoffmann et al. addressed the “PEG-dilemma” in nucleic acid delivery with folic-acid functionalized cationic lipoplexes, demonstrating enhanced efficiency in delivering therapeutic nucleic acids for treating cancers (Hoffmann et al.). Additional studies revealed the anti-photoaging efficacy of C-phycocyanin (C-PC) from Spirulina on UVB-irradiated skin, using advanced nano-dispersion for transdermal delivery *in vivo* tests. Results showed reduced aging signs with better collagen structure, and balanced oxidative stress, suggesting potential natural anti-aging treatments (Zhou et al.). Concurrently, research from Liang et al. underscores nanotechnology’s impactful role in tendon repair. Given tendons’ limited natural healing, leading to scarring and reduced function, the study highlights the role of nanotechnology and nanoparticles for targeted drug and gene delivery (Liang et al.). These findings improve cancer therapeutic efficacy and illustrate the transformative potential of nanoparticles in enabling multi-faceted treatment strategies.

### 2.2 Metal-Organic Frameworks (MOFs) in drug and gene delivery

While nanoparticles have shown significant promise as a platform of drug and gene delivery, another material known as Metal-Organic Frameworks (MOFs) has emerged with potential applications that are also remarkable. With the wide range of bioactive molecules, MOFs offer controlled drug release, making them ideal for targeted therapeutic applications. Moreover, because of the high porosity, MOFs have a larger loading capacity, ensuring enhanced payload stability. Due to their customizable nature, MOFs offer targeted drug delivery and improve biocompatibility, reduce potential side effects (Lazaro and Forgan, 2019; Lawson et al., 2021). One study illustrated the potential of ZIF-8 loaded with Rutin (ZIF-8@Rutin) as a multifunctional therapy for chronic wounds infected by drug-resistant bacteria. The nanocomposite created demonstrated effective bacterial killing capabilities and promoted wound healing while maintaining acceptable cytocompatibility (Xia et al.). Another study presented Lar@Fe-MOF as a carrier for the anticancer agent, larotrectinib, showing substantial drug loading and slow-release properties, demonstrating MOFs’ suitability as carrier materials in cancer therapy (Gan et al., 2023). Both studies underscore MOFs’ potential to overcome challenges like low bioavailability and cytotoxicity and bring them possibility for wild use in medical treatments.

### 2.3 Nanofiber-based drug delivery

Beyond the significant advancements brought by nanoparticles in drug and gene delivery, nanofiber-based drug delivery systems are carving out their own niche in the realm of pharmaceuticals. A study has explored the potential of transforming liquid licorice into fast-dissolving nanofiber formats using electrospinning to address pharmaceutical challenges. This innovative nanofiber delivery system not only solves the longstanding Research Topic related to solubility and targeted delivery but also enhances palatability through the incorporation of sucralose (Liu et al.).

## 3 Bioengineering platforms for drug and gene delivery

Exploration in the realm of bioengineering technologies has brought forth revolutionary platforms essential for the optimization of drug and gene delivery. These technologies tap into the potential of biological systems and materials, thereby offering benefits like superior biocompatibility and sophisticated command over therapeutic delivery systems.

For example, small extracellular vesicles (sEVs) present a promising platform for drug loading, especially for hydrophilic drugs. Man et al. presented an “Esterase-responsive Active Loading” (EAL) method to enhance drug loading in sEVs. While sEVs show potential for drug delivery due to low immunogenicity, their loading efficiency has been a concern. The EAL method uses ferulic acid ester derivatives and enzyme-responsive mechanisms for better loading than passive methods. The research also highlights large-scale sEV production and confirms the low toxicity of EAL-sEVs. This study suggests a practical solution for efficient therapeutic delivery (Man et al.).

Building upon the foundation of bioengineering technologies, the synergy between bioengineering and nanotechnology is used wildly in the field of drug and gene delivery. Magnetoliposomes (MLPs) proposed by Cifuentes et al. is a new drug delivery system for Parkinson’s Disease (PD) treatment, merging bioengineering and nanotechnology. The system uses magnetite nanoparticles bioengineered with translocating protein (OmpA) within soy lecithin liposomes. This boosts drug bioavailability, like Levodopa, essential for PD. The study shows how bioengineering improves biocompatibility and nanotechnology refines nanoparticle design. Molecular simulations confirm OmpA’s role and tests validate MLPs’ improved biocompatibility, offering advancements in PD treatments. (Cifuentes et al.). Collectively, by harnessing biological systems and materials, these bioengineered platforms promise improved biocompatibility and finer control over therapeutic mechanisms, paving the way for advanced medical treatments.

## 4 Conclusion

In the burgeoning fields of nanotechnology and bioengineering, transformative advancements are shaping the future of drug and gene delivery systems. As these technologies continue to evolve, they promise to bring about more targeted and efficient therapeutic solutions for a range of medical conditions. However, despite substantial progress, several significant challenges persist, including the full development of scalability and reproducibility of these innovations for clinical implementations. There are also concerns about potential toxicity, immunogenicity, and long-term effects of using nanomaterials within biological systems, which will require further exploration (Patra et al., 2018).

But still, the advances in nanotechnology and bioengineering offer promising pathways to overcoming the long-standing barriers in drug and gene delivery. While challenges remain, the integration of these multidisciplinary methodologies reveals myriad opportunities to formulate more efficacious, safer, and individualized therapies, heralding a paradigm shift in the realm of precision medicine.
